# Intelligent Evaluation of Public Sports Service Based on Intuitionistic Fuzzy Set Theory

**DOI:** 10.1155/2022/4748730

**Published:** 2022-08-21

**Authors:** Yu Shao, Rong Bo

**Affiliations:** ^1^Department of Leisure Sport, Shanghai University of Sport, Shanghai 200438, China; ^2^Institute of Physical Education, Central South University of Forestry and Technology, Changsha 410004, Hunan, China

## Abstract

At this stage, in the research process of sports public service efficiency evaluation and analysis, due to the use of accurate data analysis methods, there are problems of low evaluation reliability and low analysis speed. Based on this, this paper studies the application of an information strategy based on the intuitionistic fuzzy data matching method in the evaluation and analysis of sports public service efficiency. The efficiency evaluation and analysis model of sports public service based on the intuitive fuzzy information algorithm is established. According to the characteristics of different types of sports public service, different types of data analysis methods are used to evaluate the efficiency, and the optimization of the efficiency analysis model of sports public service is realized according to the service demand and data type of different sports events. Finally, an experiment is designed to quantitatively evaluate the accuracy, stability, and reliability of the sports public service analysis model. The results show that the efficiency intelligent evaluation and analysis model based on the intuitive fuzzy information algorithm can effectively select the optimal sports public service evaluation countermeasures and rules according to the characteristics and data types of different sports events, realize the multi-dimensional accuracy classification of different types of sports events, and effectively improve the reliability of sports public service efficiency evaluation.

## 1. Introduction

Implementing the national strategy of nationwide fitness is the basic task of promoting healthy China to build a sports power. In order to optimize the transformation of government service functions, we will vigorously promote the development of public services for national fitness, so that the grass-roots people can enjoy the fruits of sports development and enhance their sense of gain and happiness. It is particularly important to improve the public sports service system and improve the efficiency of public sports service. Therefore, it is necessary to evaluate the efficiency of public sports services, explore the relevant influencing factors, solve the practical problems of the current reform and development of sports, and put forward effective paths for reference. The evaluation and analysis of sports public service efficiency has important practical significance for the improvement of public service [1]. At this stage, due to different types of sports and different service contents, it is easy to have a variety of inefficient services, which will affect the experience of public users [2]. Although there are many researches on the efficiency evaluation of sports public service at this stage, there are still no good migration application research results, and many methods still need to be analyzed in combination with the problems existing in the public service of specific sports types [3]. Therefore, in order to better develop a more targeted evaluation system based on different types of sports public service strategies, it is necessary to combine the intuitive fuzzy information algorithm to realize high-quality analysis and customized evaluation of different sports events [4].

Based on this background, this paper studies the application of the intuitionistic fuzzy information algorithm in intelligent analysis of sports public service efficiency evaluation, which is mainly divided into four chapters. The first chapter briefly introduces the application background of sports public service efficiency evaluation and intuitionistic fuzzy information algorithm and the chapter arrangement of this study. The second chapter introduces the research status of the efficiency evaluation model of sports public service and summarizes the shortcomings of the current research. The third chapter constructs the evaluation and analysis model of sports public service efficiency based on the intuitive fuzzy information algorithm. Through the disturbing intelligent analysis of different types of sports service data, it realizes the high-intensity representation of its internal relevance and carries out centralized control according to its internal error to improve the accuracy of the model. Chapter 4 tests the innovative characteristics of different types of data groups of the intelligent analysis sports public service efficiency evaluation model constructed in this paper and realizes the accuracy verification and analysis of the sports public service efficiency model by analyzing the error degree of the efficiency evaluation process of different types of sports public services. The experimental results show that compared with the traditional sports public service analysis and matching model of database modular analysis and processing, the sports public service efficiency evaluation and analysis model based on the intuitive fuzzy information algorithm proposed in this study has better use value and lower error rate.

The innovation of this paper is to propose an efficiency evaluation and analysis model of sports public service based on the intuitionistic fuzzy information algorithm. The model can evaluate the accuracy of different data groups according to the intelligent degree of sports public service efficiency evaluation types corresponding to different types of sports events and the efficiency analysis according to the intelligent classification effect of data, in order to improve its internal data accuracy.

## 2. State of the Art

At present, many mathematicians have carried out a lot of research according to the efficiency of sports public service, mainly focusing on service data group, innovative group analysis, and efficient intelligent matching and tracking [5]. Researchers Crothers and others adopted different matching strategies and data analysis stability thinking for different databases according to different types of sports, so as to realize intelligent and stable tracking of different data groups, and their internal data relevance has good analysis and fuzzy rules, so they can better complete the efficiency analysis of different sports public services. However, more parameters need to be checked [6]. Ni and other scholars adopted different types of data group classification ideas for different sports public service methods from the differences of service strategies and service methods of different types of sports, tree thinking strategy, and grid management thinking at marriage [7]. In the process of analyzing the efficiency evaluation of sports public service, Zhao and other scholars found that different types of sports have different sets of data centers, so they adopted a source efficient attack model to realize targeted analysis and intelligent matching and tracking of data groups [8]. According to the characteristics of fuzzy information processing system, Rahimian and other scholars intelligently analyze different types of sports public service methods, classify different data, and use repair sparsity algorithm for in-depth analysis [9]. Simone and other scholars adopted an intelligent adaptive allocation strategy for sports events according to different types of sports service thinking. The experimental results show that this strategy can carry out feature recognition and intelligent analysis according to its internal differences and can be used for optimal matching analysis of different types of strategy groups [10]. Wu and other scholars conducted targeted analysis on the service efficiency of different sports events according to the differentiated characteristics of power points of sports events, discussed their internal relevance of sports events and evaluated their efficiency, and proposed a sports public service analysis model based on multi-dimensional innovation matching identification [11]. Relying on different types of high-end sports public service databases, Yang and other scholars classify the efficiency of sports public service for different types of sports events and put forward a sports public service analysis model that can match high-value data [12]. In the process of studying the efficiency evaluation of sports public service, Pradeep and other scholars found that different types of efficiency analysis problems need different methods for stability matching. Therefore, they proposed a high-intensity and multi type collaborative sports public service analysis and efficiency stability evaluation system. The system can effectively improve the service efficiency and data accuracy of different types [13].

To sum up, it can be seen that the current commonly used intelligent analysis models for sports public service efficiency evaluation cannot efficiently complete the quantitative analysis of high-precision data of sports events and need to classify and analyze the innovation of different model data. Therefore, there is the possibility of further research on the efficiency and stability of data analysis [14–16]. On the other hand, in the research on the differentiation of sports public service efficiency evaluation, there are few research results combined with the intuitive fuzzy information algorithm and few unified analysis combined with the fuzzy information strategy [17–19]. Therefore, it is of great significance to carry out the model analysis and research based on the fuzzy information algorithm applied to the efficiency evaluation of sports public service.

## 3. Methodology

### 3.1. Analysis of Intelligent Analysis Model for Efficiency Evaluation of Sports Public Service

**Figure 1:**
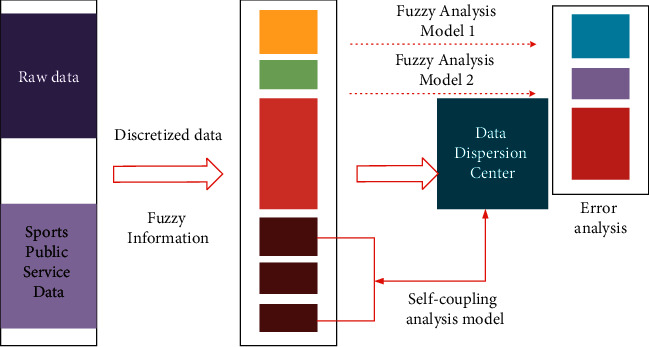
Data operation and processing process of intuitionistic fuzzy information algorithm.

Intuitionistic fuzzy information theory adopts the discrete processing technology of data group and multi-dimensional data matching and tracking strategy to realize data analysis and processing and intensity matching to varying degrees [20]. The current data analysis types have different degrees of error, which is because the parameters set by different data analysis types have many differences. Therefore, in the process of solving specific problems, the differences of parameters are very different, which will affect the final data operation results [21]. Intuitionistic fuzzy information algorithm is mainly used to quantitatively analyze the targeted data of different sports public service efficiency and evaluation in solving the problem of sports public service efficiency evaluation. Through the value matching of different data groups, it can classify different types of data groups and then realize the high-accuracy analysis of different service efficiency [22]. The thinking process of data operation is shown in Figure 1. On the other hand, in the process of differential analysis of different types of sports public service projects, there will be great differences in their internal data matching degree and value analysis strategy, so there will be obvious differences in their internal data analysis intensity, and there is great uncertainty in their internal data accuracy [23]. In the process of adopting diversified data analysis dimension strategy for the data type of sports public service efficiency evaluation, the value degree will be matched according to its internal “Anjian performance,” so as to realize the classification and quantitative research of the data group [24]. By using the intuitionistic fuzzy information algorithm and computer network analysis technology, the difference characteristics of its internal data types can be well analyzed, especially in value matching and stability strategy [25].

### 3.2. The Process of Establishing the Efficiency Evaluation Model of Sports Public Service

After the analysis and intelligent evaluation of different types of sports public service efficiency, high-precision quantitative analysis and stability solution can be realized according to its external relevance and internal matching. Usually, the quantitative analysis of sports public service mainly adopts common statistical analysis. However, it is difficult to obtain data related to sports public services or because they are often mixed with public services. Therefore, the relevant statistical analysis is difficult, even difficult to carry out. Compared with a large number of mathematical models and quantitative analysis methods and means in the fields of economy, management, education, and so on, it is not common to adopt similar methods in the field of sports public service.

**Figure 2:**
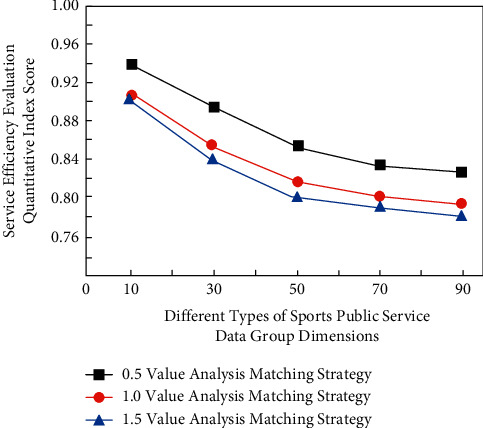
Data simulation analysis process based on computer intuitive fuzzy high latitude value analysis strategy.

Therefore, after the simulation analysis of different types of sports public service efficiency strategies combined with the fuzzy information classification algorithm, the data simulation analysis process based on computer intuitive fuzzy high latitude value analysis strategy is shown in Figure 2.

It can be seen from Figure 2 that for the sports public service efficiency evaluation data sets with different value analysis matching strategies, the evaluation scores of different types of sports public services are different because different types of fuzzy information data analysis networks and intuitive fuzzy algorithms are in the process of high-precision analysis of the data group. Eight dimensional matching classification will be carried out according to the accuracy of its data group to realize the classified processing of different service events, and then value analysis will be carried out according to its internal error, and its efficiency will be evaluated. In this process, the corresponding evaluation function *P*(*x*) and efficiency function *G*(*x*) can be expressed as *h*_*c*_=1/*M* × *N*∑_*i*=0_^*M*−1^∑_*j*=0_^*N*−1^*δ*(*f*_*ij*_ − *c*).

(1) $$\begin{array}{c} P\left(x\right)=\frac{8\sum_{i=1}^{n}\left(x_{i}^{2}+x_{i-1}^{2}/2\right)}{3x_{i-1}^{2}+5x_{i-2}^{2}}, \end{array}$$

(2) $$\begin{array}{c} G\left(x\right)=\frac{\sqrt{\sum_{i=1}^{n}\left(x_{i}^{2}+x_{i-1}^{2}/2\right)/3x_{i-1}^{2}+4x_{i-2}^{2}}}{8\sqrt{x_{i}^{2}+x_{i-2}^{2}}}. \end{array}$$

The solution expression *L*(*x*) of the eigenvector corresponding to different public sports service data groups is

(3) $$\begin{array}{c} L\left(x\right)=\sqrt{\frac{\underset{x⟶\infty }{\lim}\sqrt{G{\left(x\right)}^{2}+P\left(x\right)}}{\underset{x⟶0}{\lim}\sqrt{G{\left(x\right)}^{2}+rP\left(x\right)}}}, \end{array}$$

where *r* is the high-value response value of public service efficiency evaluation, which is converted into the classified value parameter of high latitude, then the corresponding discrete function *R*(*x*) formula is expressed as

(4) $$\begin{array}{c} R\left(x\right)=\frac{\underset{x⟶\infty }{\lim}G\left(x_{k}\right)+x_{k}}{L\left(x_{k}\right)}, \end{array}$$

where *x*_*k*_ represents the low latitude parameter of public sports service. Complete different types of arrays and the corresponding combination strategy formula is

(5) $$\begin{array}{c} R\left(x_{k+1}\right)=\frac{\sqrt{R{\left(x_{k}\right)}^{2}+L{\left(x_{k}\right)}^{2}}}{L\left(x_{k-1}\right)}. \end{array}$$

**Figure 3:**
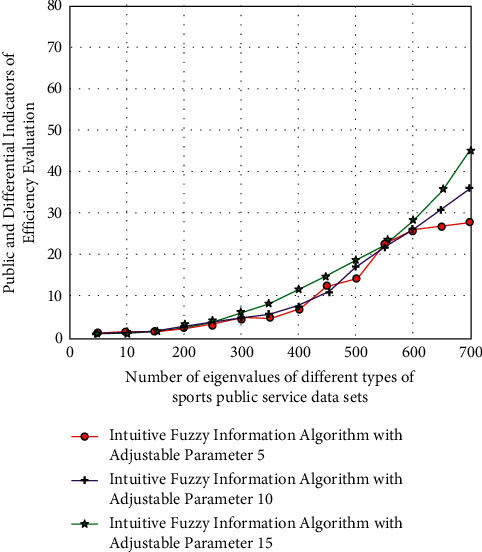
Analysis results of high-value function based on intuitive fuzzy evaluation algorithm.

After discarding the correlation degree of its internal data type, with the help of the intuitive fuzzy strategy, its corresponding efficiency classification function is

(6) $$\begin{array}{c} U\left(x_{k}\right)=8\sqrt{\frac{\sqrt{R{\left(x_{k}\right)}^{2}+L{\left(x_{k}\right)}^{2}}}{8R\left(x_{k}\right)+3L\left(x_{k-1}\right)}}, \end{array}$$

where *x* is the high-intensity-type parameter group of the intuitionistic fuzzy classification. After completing this link, the corresponding high-value degree function analysis results are shown in Figure 3.

According to the results of Figure 3, after classifying the efficiency evaluation data of sports public service by using the intuitive fuzzy information algorithm, different types of arrays have great change intensity, and their efficiency is also different and multidimensional. In essence, public service efficiency is a production efficiency problem within the public service system. Most of the current research methods use the nonparametric method to determine the frontier production function, that is, the data envelopment analysis (DEA) method for research evaluation. It is a nonparametric linear mathematical programming method for convex efficient frontier estimation. At present, this method has been widely used in the efficiency evaluation and research of various industries and fields. This is because different types of fuzzy information analysis algorithms can realize high-dimensional classification and evaluation analysis of data according to super-precision function group. Then it realizes the quantitative representation and value matching analysis of the efficiency evaluation of sports public service.

**Figure 4:**
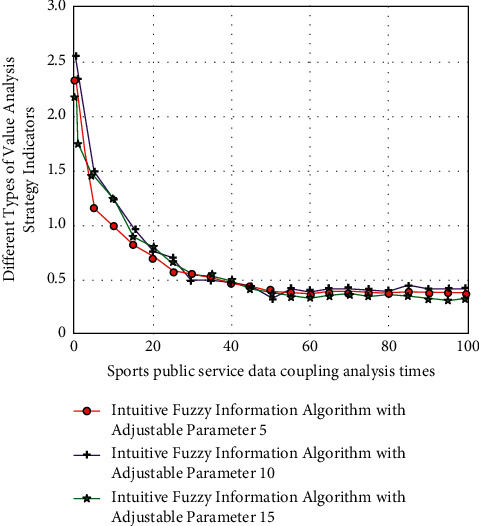
Simulation analysis results of sports public service efficiency indicators based on intuitionistic fuzzy information.

Set the basic analysis value of the sports public service efficiency of the corresponding intuitive fuzzy information in this study as 0.1, and the simulation analysis results are shown in Figure 4.

It can be seen from Figure 4 that when characterizing and intensity matching analysis of sports public service efficiency evaluation data, the internal relevance data groups also have different types of value analysis strategies because different types of data groups need to carry out value matching and dimension correspondence in combination with the types of data groups in value analysis. At this time, the corresponding intuitionistic fuzzy information function can be expressed as

(7) $$\begin{array}{c} r\left(x\right)=\sqrt{\frac{w\sqrt{x}+bx}{w+b}}. \end{array}$$

The corresponding application restrictions are

(8) $$\begin{array}{c} \frac{x_{i}\left[h\left(x\right)+n\right]}{w\sqrt[{3}]{w}+r\sqrt[{3}]{b}}\geq \frac{x_{i}\left[h\left(x\right)-n\left(x\right)\right]}{w\sqrt[{3}]{w}+r\sqrt[{3}]{b}}. \end{array}$$

Under the intuitionistic fuzzy analysis algorithm, the corresponding truth function is *T*(*x*):

(9) $$\begin{array}{c} T\left(x\right)=\sum_{i=1}^{n}rx_{i}+b\left(x\right), \end{array}$$

where *b*(*x*) represents the truth value correspondence analysis function, *w* is the real array of sports public service efficiency evaluation, and *b* is the two-dimensional editing number of sports public service efficiency evaluation data group.

In order to improve the accuracy of its evaluation efficiency, it is also necessary to add the necessary conditions for significance classification:

(10) $$\begin{array}{c} \frac{x_{i}\left[h\left(x\right)+n\right]-x_{i-1}}{b\sqrt{x_{i}}}>b\sqrt{x_{i-1}+x_{i}}. \end{array}$$

Under this significant condition, the formula corresponding to the collimation function *β*(*w*, *x*) is

(11) $$\begin{array}{c} \beta \left(w,x\right)=\frac{\sqrt{{\left\|w^{2}\right\|}^{3}+2/3\left(K-x\sum_{i=1}^{n}x_{i}\right)}}{b\left(w,x\right)}, \end{array}$$

where *K* represents the standard threshold function of intuitionistic ambiguity.

In order to further improve the standardization of fuzzy information, the gradient function *H*′(*x*) of the judgment function in different dimensions is

(12) $$\begin{array}{c} H^{\prime }\left(x\right)=\frac{\sqrt{\left|\sum_{i=1}^{n}a_{i}x_{i}\left(x_{i}\times x\right)+b\left(x_{i}\times x\right)\right|}}{H\prime \left(x-1\right)}, \end{array}$$

(13) $$\begin{array}{c} H^{\prime \prime }\left(x\right)=\frac{\sqrt{\left|\sum_{i=1}^{n}a_{i}x_{i}\left(x_{i}\times x\right)+b\left(x_{i}\times x\right)\right|}}{H\prime \prime \left(x-1\right)}, \end{array}$$

(14) $$\begin{array}{c} H^{\prime \prime \prime }\left(x\right)=\frac{\sqrt{\left|\sum_{i=1}^{n}a_{i}x_{i}\left(x_{i}\times x\right)+b\left(x_{i}\times x\right)\right|}}{b\left(x_{i}\right)H\prime \prime \left(x-1\right)}. \end{array}$$

**Figure 5:**
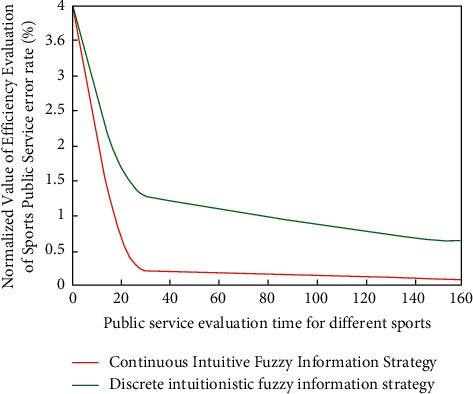
The corresponding fuzzy information analysis results under different differentiation conditions.

After quantitative evaluation, the corresponding public service efficiency analysis function is

(15) $$\begin{array}{c} Z\left(x_{j},x_{i}\right)=\frac{\underset{\delta x⟶0}{\lim}\sqrt{\left|\sum_{i=1}^{n}a_{i}x_{i}\left(x_{i}\times x\right)+b\left(x_{i}\times x\right)\right|}/b\left(x_{i}\right)H^{\prime \prime }\left(x-1\right)}{\left|x_{i}-x_{j}\right|}, \end{array}$$

where *a*_*i*_ represents data groups of different groups. At this time, the corresponding fuzzy information analysis results under different differentiation conditions are shown in Figure 5.

It can be seen from Figure 5 that when the efficiency evaluation of sports public service normalizes and analyzes different sports, the corresponding numerical changes are also quite different, but there are some regular and stable changes in local data. This is because the internal relevance is very different under the analysis of the intuitive fuzzy information algorithm. Therefore, the corresponding standardized single factor has good universality and stability.

## 4. Result Analysis and Discussion

### 4.1. Experimental Design of Sports Public Service Efficiency Evaluation Model Based on Intuitionistic Fuzzy Information Algorithm

During the experiment, in order to further confirm the credibility and feasibility of the sports public service efficiency evaluation model proposed in this study in dealing with unknown problems, it is necessary to carry out the authenticity data analysis experiment. The fuzzy evaluation analysis strategy used in this experiment is a multi-dimensional method combining fuzzy information processing and intelligent normalization strategy. In the process of analyzing different types of sports public service data groups, this method has high accuracy and periodicity in its internal relevance evaluation and value analysis. Specifically, the research selects quantifiable public service input and output variables. Generally speaking, to study the efficiency of public services between regions, we need to take all public services or some major public service projects provided by local governments as the research object to comprehensively investigate their efficiency. Here, we generally choose two main basic public service items, medical care and education, as samples for research. In terms of variable selection, in order to make the input-output data of various regions comparable, we all choose the per capita index for investigation. In terms of public service input variables, we select the per capita public service expenditure in each region, which represents the resources consumed by each region to provide public services. In terms of output variables, we selected three indicators from the fields of health care and education to reflect their comprehensive output.

**Figure 6:**
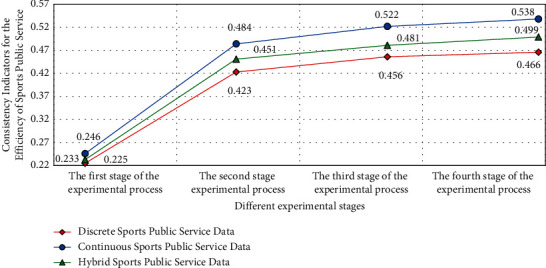
Preliminary experiment results of computer chaos algorithm analysis model.

Therefore, its internal data groups have good service characteristics and value types.  Figure 6 is the preliminary experimental results based on the intuitive fuzzy information algorithm analysis model.

In the experimental results in Figure 6, it can be found that under the analysis of different types of intuitive fuzzy information algorithms, the internal differences in data reliability and stability of different sports public service data groups are obvious, and the results have high variability and consistency in the process of discretization of sports service behavior. This result is also in line with the experimental expectations. This is because under the intuitionistic fuzzy information analysis strategy, the data calculation efficiency of the corresponding sports public service efficiency evaluation model is improved, and its internal data group and reliability will also change, which leads to higher differences in its internal relevance and matching tracking rate.

### 4.2. Experimental Results and Analysis

**Figure 7:**
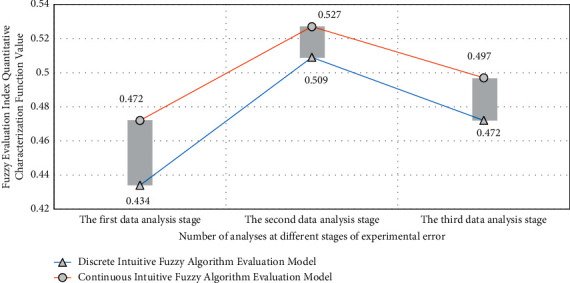
Final experimental analysis results in computer monitoring mode.

This paper evaluates the residents' satisfaction with sports public service and constructs the evaluation index system. At present, the evaluation index of sports public service should be determined from the dimensions of the construction of national fitness facilities, the construction of national fitness organizations, the development of national fitness activities, and the guidance service of national fitness. In the sports public service efficiency evaluation and analysis model based on fuzzy information processing algorithm proposed in this study, the commonly used evaluation index is used as the necessity reference strategy. If the error analysis is not carried out, the reliability of the results of the corresponding high-precision efficiency evaluation model will be reduced. Therefore, its internal relevance and data thinking coupling have different value analysis strategies, which will be directly related to the analysis accuracy of sports public service efficiency evaluation. The experimental analysis results are shown in Figure 7, in which the horizontal axis is the number of experimental analysis and the vertical axis is the quantitative characterization function value of the fuzzy evaluation index.

As can be seen from Figure 7, when analyzing different types of data groups, the experimental group using intuitionistic fuzzy strategy has the lowest error degree of corresponding data results, and its corresponding efficiency evaluation parameters are also the highest. This is because after adopting the fuzzy information processing algorithm and intuitionistic fuzzy analysis strategy, the matching degree of the corresponding data analysis and the value degree of the disturbing error function will show the characteristics of strong pertinence and high efficiency. Therefore, when the difference of the corresponding different function types in the analysis process is large, the accuracy and value degree of the corresponding evaluation and analysis vector will have good reliability and stability, and its internal relevance vector group will also have a coupling effect with the internal matching degree of the value function, resulting in higher accuracy and value matching degree in its evaluation of the efficiency of sports public service. Therefore, its evaluation results can improve the accuracy by at least 33% and be more convincing.

## 5. Conclusion

This paper studies the application of information strategy based on the intuitionistic fuzzy data matching method in the evaluation and analysis of sports public service efficiency. An evaluation and analysis model of sports public service efficiency based on the intuitionistic fuzzy information algorithm is established. According to the characteristics of different types of sports public service, different types of data analysis methods are used to evaluate its efficiency, and according to the service needs and data types of different sports events, the optimization of the efficiency analysis model of sports public service is realized. Finally, an experiment is designed to quantitatively evaluate its accuracy, and the stability and reliability of the sports public service analysis model. The results show that the effectiveness intelligent evaluation analysis model based on the intuitionistic fuzzy information algorithm can effectively select the optimal sports public service evaluation countermeasures and rules according to the characteristics and data types of different sports events, realize the multi-dimensional and accurate classification of different types of sports events, and effectively improve the reliability of sports public service efficiency evaluation.

However, this paper only focuses on the evaluation strategy of the efficiency of sports public service, so there is still room for improvement. We can conduct in-depth research on the scope of application and regional differences of the efficiency evaluation model of sports public service.

## Data Availability

The data used to support the findings of this study are available from the corresponding author upon request.
